# Effectiveness of artemether-lumefantrine provided by community health workers in under-five children with uncomplicated malaria in rural Tanzania: an open label prospective study

**DOI:** 10.1186/1475-2875-10-64

**Published:** 2011-03-16

**Authors:** Billy E Ngasala, Maja Malmberg, Anja M Carlsson, Pedro E Ferreira, Max G Petzold, Daniel Blessborn, Yngve Bergqvist, José P Gil, Zul Premji, Andreas Mårtensson

**Affiliations:** 1 Malaria Research, Infectious Diseases Unit, Department of Medicine Solna, Karolinska University Hospital, Karolinska Institutet, Stockholm, Sweden; 2 Department of Parasitology, Muhimbili University of Health and Allied Sciences, Dar es Salaam, Tanzania; 3 Division of Global Health IHCAR, Department of Public Health Sciences, Karolinska Institutet, Stockholm, Sweden; 4 Nordic School of Public Health, Gothenburg, Sweden; 5 Dalarna University College, Borlänge, Sweden; 6 Department of Physical and Analytical Chemistry, Uppsala University, Uppsala, Sweden; 7 Centre of Molecular and Structural Biomedicine, Institute for Biophysics and Biotechnology, University of Algarve, Gambelas, Portugal; 8 Laboratory of Molecular Anthropology and Health, Department of Anthropolgy, Binghamton University, Binghamton, NY 13902-6000, USA

## Abstract

**Background:**

Home-management of malaria (HMM) strategy improves early access of anti-malarial medicines to high-risk groups in remote areas of sub-Saharan Africa. However, limited data are available on the effectiveness of using artemisinin-based combination therapy (ACT) within the HMM strategy. The aim of this study was to assess the effectiveness of artemether-lumefantrine (AL), presently the most favoured ACT in Africa, in under-five children with uncomplicated *Plasmodium falciparum *malaria in Tanzania, when provided by community health workers (CHWs) and administered unsupervised by parents or guardians at home.

**Methods:**

An open label, single arm prospective study was conducted in two rural villages with high malaria transmission in Kibaha District, Tanzania. Children presenting to CHWs with uncomplicated fever and a positive rapid malaria diagnostic test (RDT) were provisionally enrolled and provided AL for unsupervised treatment at home. Patients with microscopy confirmed *P. falciparum *parasitaemia were definitely enrolled and reviewed weekly by the CHWs during 42 days. Primary outcome measure was PCR corrected parasitological cure rate by day 42, as estimated by Kaplan-Meier survival analysis. This trial is registered with ClinicalTrials.gov, number NCT00454961.

**Results:**

A total of 244 febrile children were enrolled between March-August 2007. Two patients were lost to follow up on day 14, and one patient withdrew consent on day 21. Some 141/241 (58.5%) patients had recurrent infection during follow-up, of whom 14 had recrudescence. The PCR corrected cure rate by day 42 was 93.0% (95% CI 88.3%-95.9%). The median lumefantrine concentration was statistically significantly lower in patients with recrudescence (97 ng/mL [IQR 0-234]; n = 10) compared with reinfections (205 ng/mL [114-390]; n = 92), or no parasite reappearance (217 [121-374] ng/mL; n = 70; p ≤ 0.046).

**Conclusions:**

Provision of AL by CHWs for unsupervised malaria treatment at home was highly effective, which provides evidence base for scaling-up implementation of HMM with AL in Tanzania.

## Background

Malaria still causes significant morbidity and mortality, primarily among under-five children in sub-Saharan Africa[1]. Access to prompt, effective malaria treatment within 24 hours of the onset of symptoms is critical to prevent mortality and reduce morbidity[2]. However, access to effective anti-malarial medicines and other preventive interventions is limited especially in remote rural areas of sub-Saharan Africa [3-5]. To improve early access of effective anti-malarial treatment, the World Health Organization (WHO) is promoting home-based management of malaria (HMM) [5]. This strategy involves training of community health workers (CHWs) to manage malarial illness; access to effective, pre-packed anti-malarial medicines; an effective communication strategy; and good mechanism for supervision and monitoring [2]. Previous studies have demonstrated that prompt and effective treatment of uncomplicated malaria with chloroquine at community level significantly reduced malaria-related morbidity and mortality [6-8].

Due to widespread resistance to chloroquine and sulphadoxine/pyrimethamine, most malaria-endemic countries in Africa and Asia have adopted the WHO recommendation of introducing artemisinin-based combination therapy (ACT) as first-line treatment for uncomplicated *Plasmodium falciparum *malaria [9]. Artemether-lumefantrine (AL) is presently the most widely adopted ACT in Africa, including Tanzania [10]. Although AL has a relatively complex treatment regimen, i.e. twice daily for 3 days, data from sub-Saharan Africa at health facility level indicate that AL is highly effective even with unsupervised administration [11-14]. Moreover, recent studies have shown that ACT can be successfully integrated into the HMM strategy[15-17]. These studies indicate that CHWs can safely dispense ACT with suggestible good adherence by caregivers to the correct treatment schedules. However, concerns remain among researchers and policy-makers due to limited data on the effectiveness of using ACT in the HMM strategy [18,19]. Another concern is that widespread presumptive use of ACT may spur development of parasite tolerance/resistance to these precious medicines[20].

To improve targeting of ACT to malaria infected patients parasitological confirmation is essential at community level in remote areas of sub-Saharan Africa, where a majority of fever patients seek care. Antigen-based rapid malaria diagnostic tests (RDTs) may represent an important tool to improve the diagnostic efficiency if incorporated in the HMM strategy, considering that they are easy to use and interpret and do neither require access to skilled technicians nor electricity. Previous studies have shown that RDTs can be accurately used by CHWs [21,22]. Recent community-based studies in Tanzania and Ethiopia have shown that the use of RDTs by CHWs improved targeting of AL to malaria infected patients [17,23].

This study reports data on polymerase chain reaction (PCR) corrected effectiveness of AL, when provided at community level by CHWs and used unsupervised by parents or guardians at home for treatment of uncomplicated *P. falciparum *malaria in under-five children, during an extended follow-up period of 42 days, adherence to treatment by measuring lumefantrine concentrations on day 7 after initiation of treatment, and possible selection of genetic markers associated with AL tolerance/resistance.

## Methods

### Study sites

The study was conducted between March-August 2007 in two neighbouring villages, Ngeta and Mwanabwito, in rural Kibaha District, located about 50 km west of Dar es Salaam, Tanzania. These two villages, with a total population of 4,500 people, were among five villages involved in a previous assessment of RDT use by CHWs to improve targeting of ACT at the community level[17]. The study sites were purposely selected based on presence of active CHWs, accessibility during rainy season, and within three hours drive by car from Muhimbili University of Health and Allied Sciences (MUHAS), Dar es Salaam. Malaria transmission is high, with peaks related to the rainy seasons in May-July and December-January. *Plasmodium falciparum *is the dominant parasite species. There is one dispensary in each village and most referrals are sent to Mlandizi Health Centre, situated at a distance of 9 to 10 kms from the selected villages. AL was the first-line treatment for uncomplicated malaria at the time of the trial.

### Selection and training of CHWs

After consultations with dispensary staff and village leaders, six CHWs were selected among the existing 40 CHWs in the two villages. The selection of study CHWs was based on gender balance, residential area and ability to keep records. A three-day training workshop was conducted at each village dispensary. The CHWs were trained on how to recognize symptoms of both uncomplicated and severe malaria, as well as other febrile childhood non-malaria diseases, such as pneumonia (cough with fast breathing) and acute diarrhoea, as well as in administration of AL and how to educate caregivers on the use of AL. The CHWs were also trained on how to perform and interpret RDT, i.e. Paracheck Pf^®^, according to the manufacturer's instructions (Paracheck Pf, Orchid Biomedical Systems, Goa, India) and simplified pictorial instructions (RDT job aid); prepare thick blood smears and blood spots on filter paper (Whatman 3MM) from finger prick blood samples; and filling of case record forms. Each CHW was provided with a weighing scale, a digital thermometer, pre-packed AL tablets, paracetamol tablets, a dosing chart for AL, RDTs, RDT job aid and a storage iron box. The CHWs were responsible for referral of patients and storage of the study drugs and other supplies for the study. During the study period each CHW was given a monthly allowance equivalent to 25 USD.

### Patients

Children presenting to CHWs with symptoms suggestive of uncomplicated malaria were screened for study eligibility. Patients were enrolled if they met the following inclusion criteria: age of 3-59 month; body weight of ≥5 kg; fever (≥ 37.5°C axillary) or a history of fever in the preceding 24 hours; *P. falciparum *malaria confirmed by RDT and later by microscopy; able to ingest tablets orally; able to attend stipulated follow-up visits; provision of written informed consent by a parent or guardian, and absence of any general danger signs of severe disease (convulsions, lethargic/unconscious, unable to drink/breast feed, vomiting everything). Patients with negative RDT test results and/or signs of severe disease were to be immediately referred to the village dispensary for further management.

### Study design, treatments and procedures

This was an open label, single arm prospective study, assessing PCR corrected parasitological cure rate of a six-dose treatment with unsupervised intake of AL provided by CHWs to under-five children with uncomplicated *P. falciparum *infection with an extended follow-up of 42 days. Patients fulfilling the inclusion criteria were provisionally enrolled based on a positive RDT result and provided AL treatment in standard doses according to the body weight: one tablet per dose for patients weighing 5-14.9 kg (yellow blister pack) and two per dose for 15-24 kg (blue blister pack). Every blister pack had pictures showing how the drug should be given. The first dose was given under supervision of the CHWs. If patients vomited the first dose within 30 min; administration of the full dose was repeated. The other five doses were given by the parents/caregivers at home. Standardized verbal instructions on dose, frequency and advice to combine treatment with fatty meals or breast milk were given by CHWs. Paracetamol was provided to all febrile patients. Parents or guardians were instructed to report to the CHWs or the village dispensary if the child's condition had not improved after 48 hours or if worsened at any time. Anti-malarial medicines and other drugs in the study were provided by the research project free-of-charge.

Blood smears were stained with Giemsa and read at the Department of Parasitology, MUHAS. The results were provided to the CHWs and patients within three days of sampling. Children with positive RDTs but negative blood smear results were excluded from the study and referred to the dispensary. Children with microscopically confirmed malaria were definitely enrolled. Parents or guardians were requested to bring their children back to the CHWs on days 7, 14, 21, 28, 35 and 42 or on any day that they felt ill. At each visit, children were assessed for symptoms, possible adverse events, and body temperature. Finger prick blood samples were collected for microscopy (thick blood smears) and PCR analysis (on Whatman filter paper), respectively, at day of enrolment and at each follow-up visit. In addition, the research team leader collected blood samples (100 μl taken by capillary tube) on pre-treated filter papers on day 7, which were used later for determination of lumefantrine blood concentrations. If patients did not return for scheduled follow-up visits, they were actively followed-up in their homes the next day.

Patients with recurrent infections within 14 days after initiation of AL treatment and in children who developed symptoms or signs of severe malaria during follow-up were withdrawn from the study, and referred to the village dispensary or Mlandizi health centre for rescue treatment with quinine. In children with clinical failure (non-severe malaria) after day 14 or parasitological failure day 42 after the initial therapy were retreated with AL in accordance with national guidelines [24].

### Laboratory procedures

#### Microscopy

All thick blood smears were picked up from CHWs by the research team within 24 hours after collection and stained with 5% Giemsa for 20 minutes at MUHAS. Two qualified microscopists independently read all thick blood smears. The parasite density was estimated by counting the number of asexual parasites per 200 (for gametocytes 1,000) white blood cells and calculating parasites per μL, assuming a white blood cell count of 8,000 per μL. A smear was declared negative if no asexual parasites were seen after examining 200 high power fields. Disagreement in readings (positive versus negative) or an at least two fold difference in parasite density, a third decisive microscopy reading, again blinded was to be done.

#### Molecular analysis

Distinction between recrudescence and reinfection events was performed by the use of stepwise genotyping of the *P. falciparum *genes merozoite surface protein-2 (*msp2*), glutamate-rich protein (*glurp*) and *msp1 *using standard protocols [25]. Recrudescences were defined as samples containing at least one matching allelic band in all markers, from paired samples collected on day of enrolment (D0) and day of recurrent infection (R0). A reinfection was defined as the absence of any matching allelic band in at least one marker in the paired blood samples.

Single nucleotide polymorphisms (SNPs) in the *P. falciparum *chloroquine resistance transporter gene *(pfcrt*) K76T and *P. falciparum *multidrug resistance gene 1 *(pfmdr1*) N86Y, previously associated with quinoline resistance, were analysed according to established *Apo*I restriction enzyme-based PCR restriction fragment length polymorphism (PCR-RFLP) protocols [26].

#### Determination of blood lumefantrine concentrations

Capillary blood samples (100 μL, taken by heparinized micropipette) were applied on filter papers pre-treated with 0.75 M tartaric acid. These blood samples were dried, put in small zipped plastic bags and frozen at -20°C the day after sampling. After completion of the field trial they were transported to the Bioanalytics and Pharmacokinetics laboratory of Dalarna University, Sweden, where lumefantrine blood concentrations were measured by solid-phase extraction and liquid chromatography [27].

### Outcome measures

The day-42 PCR corrected parasitological cure rate was the primary efficacy outcome, i.e. proportion of patients with clearance of asexual parasitaemia within seven days of initiation of treatment, without recrudescence within 42 days after initiation of treatment, and without use of rescue medication for clinical signs of malaria. Secondary outcomes included PCR corrected parasitological cure rates on days 14 and 28 after treatment; risk of reinfections after treatment; day 7 blood lumefantrine concentrations; selection of genetic markers (SNPs) in *pfcrt *and *pfmdr1*; and adverse events defined as signs and symptoms that occurred or increased in severity after treatment started.

### Statistical analysis

Assuming that AL treatment provided by CHWs would result in less than 85% PCR corrected parasitological cure rate by day 42. According to the WHO protocol [28], with an estimated treatment failure lower than 15%, confidence interval of 95%, a precision of 10% and a 20% drop out rate, the minimum sample size needed was at least 60 patients. To compare effectiveness data from this study with both efficacy and effectiveness data from a separate study conducted at health facility level[29], a total of 200 under-five children were recruited.

Data were double-entered and validated using EpiData software, version 3.02 (EpiData Association, Odense Denmark) and analysis was performed using Stata 10.0 (StataCorp, College Station, Texas, USA). Proportions were compared with X^2 ^test or Fisher's exact test as appropriate. All patients with microscopically confirmed *P. falciparum *malaria who had taken at least one full dose of the study medication and had at least one post-baseline efficacy assessment were included in the analyses. Cure rates and cumulative proportion of recurrent parasitaemia were estimated by Kaplan-Meier survival analysis. Data were censored for patients who were lost to follow-up or for patients with reinfections, and indeterminate or missing PCR results at the last day seen.

### Ethical clearance

The study was approved by the National Institute for Medical Research in Tanzania and Regional Ethics Committee Stockholm, Sweden, and was registered with identifier NCT00454961[30]. Before enrolment, written informed consent was obtained from the parents or legal guardians of the children.

## Results

**Figure 1:**
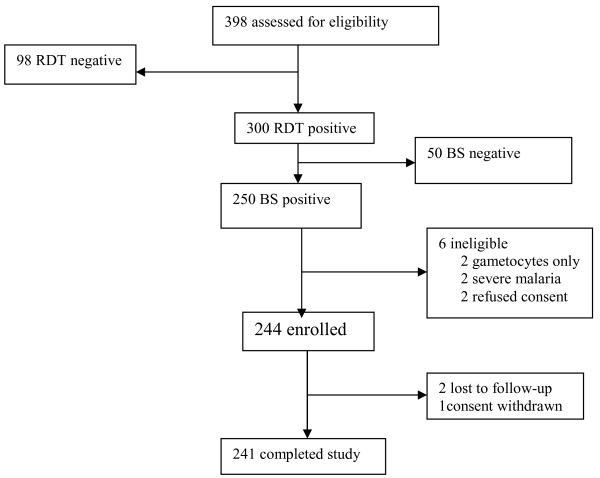
Flow of the patients through the study

Between March and July 2007, 398 febrile children were screened for study eligibility, 300 (75.4%) were provisionally enrolled based on a positive RDT, of whom 250 (83.3%) were *P. falciparum *positive by microscopy (Figure 1). Of these 250 patients, 244 (97.6%) met the inclusion criteria and were thus definitely enrolled (Figure 1). Three children (<2%) did not complete the 42-day follow-up period (Figure 1); one due to withdrawn consent on day 21 and two children moved from the study area on day 14. Baseline demographic characteristics are shown in Table 1. A majority of patients, 216/244 (88.5%), had parasite density ≥2,000/μL blood, and one child had parasite density >200,000/μL, but had no general danger signs indicating severe disease.

**Table 1:** Baseline demographic characteristics

Characteristic	(N = 244)
Male	131 (53.7%)
Age, median months (range)	31.3 (6.0-62.0)
Weight, median kg (range)	11.7 (6.0-23.0)
Number of children weighing 5-<15 kg	190 (77.9%)
Number of children weighing 15-<25 kg	54 (22.1%)
Asexual parasitaemia, geometric mean parasite/μl (range),	19054 (400-240000)
Number of patients with parasite density ≥ 2000/μl	216 (88.5%)
Temperature, °C, mean	38.0
Gametocyte carriage	8 (3.3%)
Vomiting on day 0	89 (36.5%)

### Effectiveness assessment

**Figure 2:**
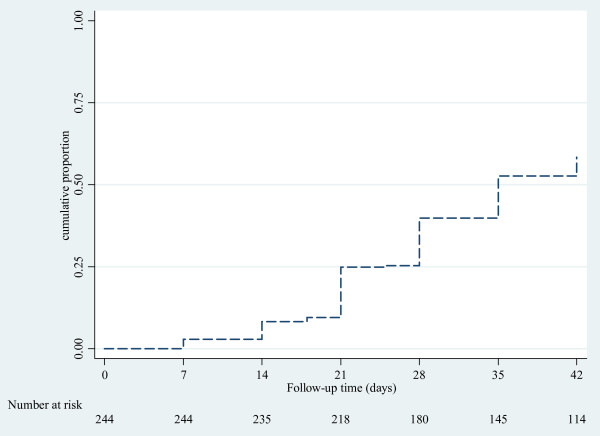
Kaplan Meier curve showing cumulative proportion of children with recurrent parasitaemia during follow-up after artemether-lumefantrine treatment

No early treatment failure was reported. On day 7, all patients were afebrile, but 7/244 (2.9%) were still parasitaemic. PCR analysis of these seven patients revealed four reinfections, two recrudescences and one indeterminate result. By day 42, some 141/241 (58.5%) patients had recurrent parasitaemia (Figure 2), of whom 32 (22.7%) presented with fever. Parasite genotyping showed that 118 (84%) were due to reinfection, 14 (10%) recrudescence and 9 (6%) indeterminate results. The PCR corrected cure rates at days 14, 28 and 42 were 97.9%, 95.1% and 93.0%, respectively (Table 2). There was no statistically significant difference in median (range) time to recrudescent infections, i.e. 21 (7-42) days compared to 28 (7-42) days for reinfections (p = 0.41). Three (21%) of the 14 children with recrudescent infection were identified after 28 days of follow-up. At baseline, eight patients carried gametocytes (Table 1). During follow-up only one gametocyte carrier was observed on day 7.

**Table 2:** PCR uncorrected and corrected cure rates as estimated by Kaplain-Meier analysis

Cure rate	rate, % (95%CI)
Day 14 PCR uncorrected	91.8 (87.5-94.6)
Day 14 PCR corrected	97.9 (95.1-99.1)
Day 28 PCR uncorrected	60.2 (53.7-66.1)
Day 28 PCR corrected	95.1 (91.4-97.3)
Day 42 PCR uncorrected	41.5 (35.3-47.7)
Day 42 PCR corrected	93.0 (88.3-95.9)

### Day 7 lumefantrine concentrations

**Figure 3:**
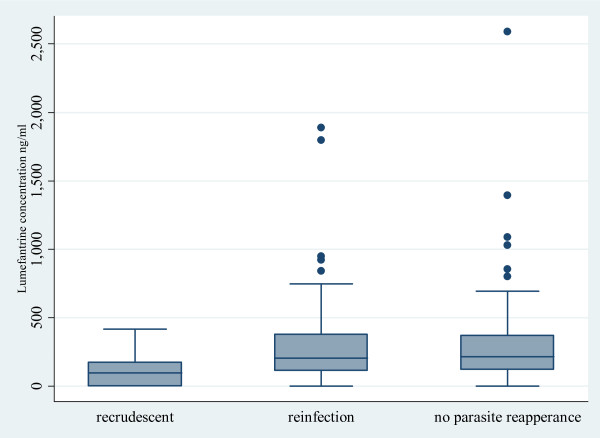
Box plots of day 7 lumefantrine concentrations versus endpoints after treatment with artemether-lumefantrine

A total of 177 of 244 (73%) patients had blood lumefantrine concentrations measured on day 7 after initiation of treatment. The median (range) lumefantrine concentration on day 7 was 205 ng/mL (0-1887). The median lumefantrine concentration was significantly lower in patients with recrudescence (97 ng/mL [IQR 0-234]; n = 10) than in those with reinfections (205 ng/mL [114-390]; n = 92), or no parasite reappearance (217 [121-374] ng/mL; n = 70; p ≤ 0.046) (Figure 3). Overall, 63% (112/177) of patients had lumefantrine concentrations <280 ng/mL[31]. Of the 10 patients with recrudescent infections analysed, eight had lumefantrine concentrations <280 ng/mL. All seven patients with parasitaemia on day 7 had lumefantrine concentration <280 ng/mL.

### *Pfmdr1 *N86Y and *pfcrt *K76T SNPs

Proportions of parasites carrying *pfmdr1 *N86 and *pfcrt *K76 at baseline and recurrent parasitemia are presented in Table 3. The *pfmdr1 *86N allele was observed to be selected among recurrent infections (p < 0.01), while the *pfcrt *K76T SNP did not show any pattern of selection upon AL administration.

**Table 3:** Frequencies of the analyzed single-nucleotide polymorphism (SNPs), before treatment (D_0_) and in recurrent infections after treatment with artemether-lumefantrine (R_0_)

		Frequencies, (pure+mix)/total*, % (95%CI)			
		*pfmdr1 *N86		*pfcrt *K76	
D_0_					
	All patients	(115+56)/234	73.1 (67.4-78.8)^a^	(152+41)/232	83.2 (78.3-88.0)
	Patients with recrudescence during follow-up	(5+8)/13	100.0 (100.0-100.0)	(7+5)/13	92.3 (75.6-100.0)
	Patients with reinfection during follow-up	(53+33)/115	74.8 (66.7-82.8)^b^	(71+23)/113	83.2 (76.2-90.2)
R_0_					
	All patients	(99+30)/138	93.5 (89.3-97.7)^a^	(92+15)/126	84.9 (78.6-91.3)
	Patients with recrudescence	(12+2)/14	100.0 (100.0-100.0)	(9+2)/13	84.6 (61.9-100.0)
	Patients with new infection	(79+25)/112	92.9 (88.0-97.7)^b^	(74+13)/104	83.7 (76.4-90.9)

* denotes the total no of successful analyses; CI, confidence interval

* denotes the total no of successful analyses; CI, confidence interval

^a^Statistically significant increase between baseline and all recurrent infections, *P *< 0.0001

^b^Statistically significant increase between baseline and only reinfections, *P *= 0002

### Adverse events

AL was well tolerated. No deaths occurred. Two serious adverse events were reported, both were due to severe malaria on days 14 and 42. In total 162 adverse events (mild or moderate in severity) were reported during follow-up, the most common being fever (34%), cough (34%) and diarrhoea (12%). These events were considered unrelated to AL treatment.

## Discussion

The results from this study showed that intake of unsupervised AL used in HMM was highly effective and well-tolerated for the treatment of acute uncomplicated *P. falciparum *malaria in Tanzanian children below five years of age. This study is the first to assess the PCR corrected cure rate achieved with ACT in the context of HMM after an extended follow-up to 42 days. A previous multicentre study showed high effectiveness of ACT used in HMM [19]. However, this study had only 28 days follow-up. Importantly, the results are consistent with previous effectiveness studies conducted at health facility level in sub-Saharan Africa [11-14].

However, a concern is the limited post-treatment prophylactic effect of AL in this high malaria transmission area, which resulted in more than half of the patients having recurrent infections within the 42-day follow-up period. A great majority of all recurrent infections were due to reinfections, when assessed with stepwise genotyping using three polymorphic genetic markers. Young children with little or no immunity to malaria may have a shorter post-treatment prophylactic effect compared to older children and adults [32]. Similar high risk of recurrent infections after AL treatment has been reported in other high transmission areas in sub-Saharan Africa [33-36]. This highlights the need of integrated approaches for malaria control. Recent data indicate that the use of ACT, supported by vector control interventions including long-lasting insecticide-treated nets (LLINs) significantly reduced malaria-associated morbidity and mortality in seven malaria-endemic countries, including Zanzibar islands in Tanzania [37].

Adherence to AL treatment in this trial was assessed by measuring day 7 lumefantrine levels, a potentially more reliable method of measuring adherence to treatment than the use of questionnaires, which has a potential for recall bias [38,39]. The method applied in this study has shown adequate sensitivity for quantification of lumefantrine from capillary blood samples collected on pre-treated filter paper[27]. Its minimal invasiveness and overall simple sampling procedure makes it a feasible field method in clinical trials conducted in remote areas. The observed median day 7 lumefantrine concentration of 205 ng/mL was similar to the concentrations achieved with unsupervised intake of AL in two recent health facility based studies conducted in the neighbouring Bagamoyo District and Malawi[29,38]. Moreover, the proportion of children (over 60%) that had a day 7 lumefantrine concentration <280 ng/mL, a cut-off level previously associated with increased risk of treatment failure [31], was similar to unsupervised groups at the health facility based studies [11,40]. The absorption of lumefantrine is known to have a high variability and suboptimal drug levels could potentially result from inadequate fat intake [11,40]. Although, in this study, fat intake was not assessed, caregivers were encouraged to accompany each AL dose with milk or fat-containing food. Low blood lumefantrine concentrations increase the risk of treatment failure and reinfections [40-43]. In this study all recrudescent infections and reinfections identified on day 7 occurred in patients with lumefantrine concentration <280 ng/mL. Similarly, a majority of patients with recrudescent infections during the 42 days follow-up had day 7 blood lumefantrine concentration <280 ng/mL. To improve adherence to unsupervised AL treatment therefore represents a key challenge for malaria control programmes in Africa to reduce the risk of adverse treatment outcome in children with uncomplicated malaria infection.

In high transmission areas, residual low/sub-therapeutic concentrations of the long acting partner drug in ACT create strong selective pressure for development and spread of tolerant/resistant parasites [44,45]. In this study, similarly to previous observations, there was significant selection of the *pfmdr1 *N86 allele in recurrent infections after AL treatment [46-50]. This data further support the potential importance of this gene in parasite response to this ACT. Less clear is the involvement of the *pfcrt *K76T SNP - in contrast to a recent study conducted in Bagamoyo District, Tanzania [46], a significant selection of the *pfcrt *K76 allele in recurrent infections after AL treatment was not observed. This might be due to lack of statistical power of the present study, taking in account the high baseline prevalence of the *pfcrt *K76 (> 80%). It can also reflect that although the potential of *pfcrt *K76T to modulate is by now well supported *in vitro *[46,51], its *in vivo *importance might be less general than the observed for *pfmdr1 *86N, being more dependent on the overall genetic makeup of the studied parasite populations.

Selecting study sites that were geographically accessible within three hours by car from Dar es Salaam might have introduced a selection bias. However, the selected villages were not different from those not included in the study in terms of number and quality of CHWs. Moreover, the weekly follow-up and supervision by researchers may have influenced the behaviour of CHWs and caregivers and resulted in improved adherence, however, supervision and monitoring of the community activities is one of the key components of the HMM strategy.

## Conclusions

In conclusion, this study showed high PCR corrected effectiveness of AL used in HMM during an extended follow-up of 42 days, which provides evidence for scaling-up implementation of HMM strategy with AL in Tanzania. However, the risk of recurrent infections after AL treatment was high, why integration of ACT with preventive interventions is critical for improved malaria control. The *in vivo *selection of genetic markers associated with drug resistance confirms that AL is vulnerable to selection of resistance-related polymorphisms in areas of high malaria transmission. It is crucial that the HMM strategy incorporates components for monitoring adherence to ACT treatment and drug resistance.

## Competing interests

The authors declare that they have no competing interests.

## Authors' contributions

BEN, ZP and AM conceived and designed the study and contributed to implementation of field study. BEN and ZP supervised the field work. MM, AMC, PEF and JPG performed molecular analyses, DB and YB performed drug level analysis. BEN and MGP analysed data. BEN and AM wrote the manuscript. All authors read and approved the final manuscript.
